# Asymmetry of the pelvis in Polish young adults

**DOI:** 10.3389/fpsyg.2023.1148239

**Published:** 2023-03-22

**Authors:** Karol Bibrowicz, Tomasz Szurmik, Katarzyna Ogrodzka-Ciechanowicz, Zuzana Hudakova, Bartłomiej Gąsienica-Walczak, Piotr Kurzeja

**Affiliations:** ^1^Science and Research Center of Body Posture, Kazimiera Milanowska College of Education and Therapy, Poznan, Poland; ^2^Faculty of Arts and Educational Science, University of Silesia, Cieszyn, Poland; ^3^Institute of Clinical Rehabilitation, Faculty of Motor Rehabilitation, University of Physical Education, Krakow, Poland; ^4^Faculty of Health, Catholic University, Ružomberok, Slovakia; ^5^Department of Health Care Studies, College of Polytechnics, Jihlava, Czechia; ^6^SNP Central Military Hospital, Faculty Hospital, Ružomberok, Slovakia; ^7^Institute of Health Sciences, Podhale State College of Applied Sciences, Nowy Targ, Poland

**Keywords:** diagnostics, human body, physiotherapy, pelvic girdle, postural asymmetry factor

## Abstract

**Introduction:**

Symmetry is one of the criteria of correct body posture in upright position. The spatial positioning of the pelvic girdle is crucial to it. Functional and structural asymmetries within the lumbo-pelvic-hip complex can have a significant influence on the structure and functions of many human body organs and systems. The aim of the study was to present the results of inclinometer measurements of selected landmarks of the pelvic girdle in young adults aged 19–29.

**Methods:**

The analysis of occurrence of spatial pelvic asymmetry was based on the authors’ original, clinical classification and the significance of the body mass and height for the analyzed asymmetries. The inclinometer measurements of the selected landmarks of the pelvic girdle were performed in a sample consisting of 300 young individuals. Then, the occurrences of the spatial asymmetry of the pelvis were analyzed based on the authors’ own clinical classification using alignment symmetry of the iliac crests, the anterior superior iliac spines and the trochanters major as a criterion. All study subjects with asymmetry <1 degree were treated as those with a symmetrical pelvis.

**Results:**

The significance of gender, body mass and height for the analyzed asymmetries was assessed. Symmetric positioning of the iliac crests was observed in only 32% of the respondents. The iliac crest depression on the left side was more frequently observed – in 41% of the respondents. This occurred more often in women (44%) than in men (38%). In the group of women, the rotated pelvis was the most often observed (39.4%) asymmetry, while for men, it was the oblique pelvis (40%). More detailed analysis by pelvic asymmetry subtypes showed their statistical differentiation between women and men (*p   * <     0.0001). Analysis of moderate rotation of the pelvis for men, were reported slightly higher values but these differences were not statistically significant (*p* = 0.253). Women, in turn, showed slightly higher mean values but here too, the differences were not statistically significant (*p* = 0.245).

**Discussion:**

Asymmetries in the pelvis area are common; they were observed in less than three-quarters of the examined population. Oblique pelvis was found in less than a quarter of women and in more than one-third men with the predominant structural asymmetries. Rotated pelvis was observed in more than one-third of women and men with dominating functional asymmetries. There were no linear correlations between the body mass and height, and the angle of asymmetries.

## Introduction

Symmetry is one of the criteria of correct body posture in upright position. Human posture in the frontal and transverse plane should show a symmetric positioning of the pelvis, straight spine and symmetric alignment of the selected landmarks in the torso area. However, it is known that in reality, this happens extremely rarely. There are many signs of asymmetry. From functional asymmetries resulting, for example, from the lateralization process, performing asymmetric movements, changes of the functional condition of muscles, tendons and joint capsules, to static asymmetries which occur in response to developmental disorders or diseases. Spatial position of the pelvic girdle is crucial for the development of correct body posture (Applebaum et al., 2021). Pelvis is an important structure which connects the torso and the lower limbs to support and transfer the load on to the legs during different functional movements. It is a part of the lower torso in the sitting position but during standing or walking, it becomes a functional element of a lower limb (Murray et al., 2017; Dubey et al., 2018).

That is why changes in pelvis position when standing affect also balance and functionality (Gurney, 2002; Verheyden et al., 2006; Khamis and Carmeli, 2017; Murray et al., 2017) In addition, control of pelvis alignment is necessary to produce more efficient movements and walking (Gurney, 2002; Staszkiewicz et al., 2012), and if it is not controlled properly during walking, the speed, stability and efficiency of the walk decreases (Dickstein and Abulaffio, 2000; Gurney, 2002; Chen et al., 2003). It is assumed that pelvis should be positioned symmetrically. However, asymmetries in its area are often observed. It is related to the anatomy as such, different bone anomalies (e.g., hip dysplasia; Li et al., 2016; Wells et al., 2017), degenerative or inflammatory processes, fractures or injuries of the pelvis (Garvey and Hazard, 2014; Tile et al., 2015; Rommens and Hofmann, 2017; Stover et al., 2017). Pelvic asymmetries most often mentioned in clinical practice include asymmetries in the frontal plane (pelvic obliquity) associated with actual or functional leg length inequality (Brady et al., 2003; Applebaum et al., 2021), asymmetries in the transverse plane (rotated pelvic), or mixed forms (Juhl et al., 2004; Al-Eisa et al., 2006; Gnat and Bialy, 2015). Asymmetries of the pelvis can be associated with the development of non-specific chronic low back pain, caused by incorrect mechanical load on the body which increases the stress on the soft tissues in the lumbar section (Brady et al., 2003; Sorensen et al., 2016; Azizan et al., 2018; Applebaum et al., 2021). They can also contribute to the development of functional scolioses (Ploumis et al., 2018; Kobayashi et al., 2020) or degenerative changes of joints (Harvey et al., 2010). Assessment of pelvic asymmetry can be useful in anatomic examinations, functional behavior analysis, mobility assessment, biomechanical explorations, development of implants and other clinical applications (Tobolsky et al., 2016; Osterhoff et al., 2019).

It seems, however, that despite so much attention paid to these issues, there are no clinical methods which would provide the base for more advanced diagnostics and support therapeutic decisions. At present, the most information refers to radiologic examinations of the pelvic girdle. Based on them, diagnostic schemes and therapy recommendations are developed. They are reliable, repetitive and accurate (Lee et al., 2015; Kurki, 2017; Handrich et al., 2021; Zhang et al., 2022a). Parallel to the radiologic examinations, methods based on palpation of the selected landmarks in the pelvic girdle area are used. Unlike radiology, they are less reliable and show great randomness (Stovall and Kumar, 2010; Kilby et al., 2012; Li et al., 2016; Mine et al., 2022). The result of a search for methods which would be at least partially as reliable as radiologic methods, are assessments using three-plane image analysis systems (Furian et al., 2013; Yu et al., 2020) or simple measuring devices like Palpation Meter or Duometer (Levangie, 1999; Petrone et al., 2003; Bibrowicz and Bibrowicz, 2011a, 2011b).

Up to date, to the best of our knowledge, there are no scientific reports clearly describing pelvic asymmetry in young adults. That is why the aim of the study was to present the results of inclinometer measurements of selected landmarks in the lumbo-pelvic-hip complex in young adults aged 19–29. The analysis of occurrence of spatial pelvic asymmetry was based on the authors’ original, clinical classification and the significance of the body mass and height for the analyzed asymmetries.

## Materials and methods

### Study design

This is an observational (cross-sectional) study. The study protocol follows the guidelines of the Helsinki Declaration. This study was conducted in compliance with the Strengthening the Reporting of Observational Studies in Epidemiology (STROBE) Statement: guidelines for reporting observational studies (von Elm et al., 2008). It was part of the research program “Assessment of position and functional mobility of lumbo-pelvic-hip complex and their influence on the quality of body posture, postural stability and locomotion in population of children, adolescents and adults.” The program was approved by the Bioethics Board of the Higher School of Physiotherapy in Wroclaw, approval No. 1/2010 of 10.04.2010, and was carried out in the Body Posture Scientific and Research Center in the College of Education and Therapy in Poznan. Each time, examinations were based on written consent provided by the participants.

### Setting

Examinations were carried out in 2016–2020 among the students of Kazimiera Milanowska College of Education and Therapy in Poznan and the Silesian University of Technology in Gliwice.

### Participants

The sample consisted of 300 young adults aged 19–29 years (150 women X = 21.4, SD = 3.18 and 150 men X = 21.2, SD = 3.52), selected by means of simple random sampling. They were the representative group recruited from the population of 2,131 individuals (1,321 women, 810 men).

The inclusion criteria were as follows:

- Age between 19 and 29 years;
- no visible locomotor system dysfunctions (lack of clearly confirmed health issue);
- lateralization—in order to unify the sample, only individuals with right-sided lateralization in the area of upper and lower limbs were recruited for further examinations;
- correct body mass according to Body Mass Index (BMI).

The exclusion criteria were:

- Syndromes related to the Central Nervous System (CNS) and/or locomotor system, hindering correct psychomotor development;
- disorders which potentially cause postural pathologies: genetic syndromes, hormonal disorders, neuromuscular diseases, congenial locomotor system defects;
- undermass, overmass, and obesity;
- lack of written consent to take part in the study.

### Outcome measures

**Figure 1:**
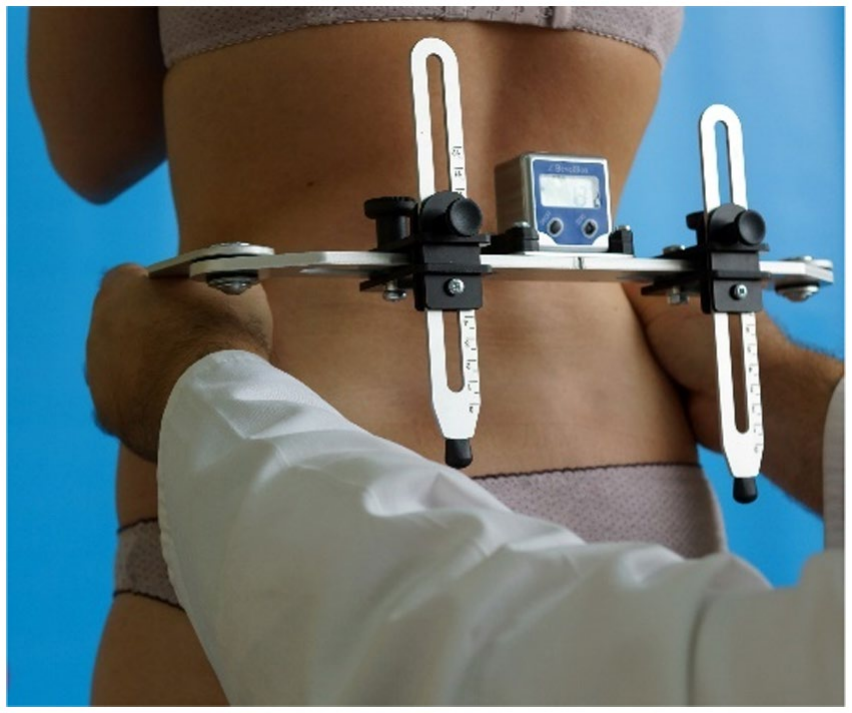
Measurement of iliac crest angle using Duometer (own source).

- Mass and height measurement (using a verified medical column scale C315.60/150.OW-3—a 100–200 cm height measuring device UNIWAG—Professional electronic scales, Krakow, Poland);
- Determination of obesity using the BMI;
- Measurement of the position of the selected landmarks in the pelvic girdle using scalable anthropometric leveler with electronic inclinometer—Duometer (Figure 1):
  - ICA—iliac crest angle—the angle between the horizontal axis and the line formed by the apices of the iliac crests;
  - ASISA—anterior superior iliac spine angle—the angle between the horizontal axis and the line formed by the anterior superior iliac spines;
  - TMA—trochanter major angle—the angle between the horizontal axis and the line formed by the tops of the greater trochanters.

### Intervention

**Figure 2:**
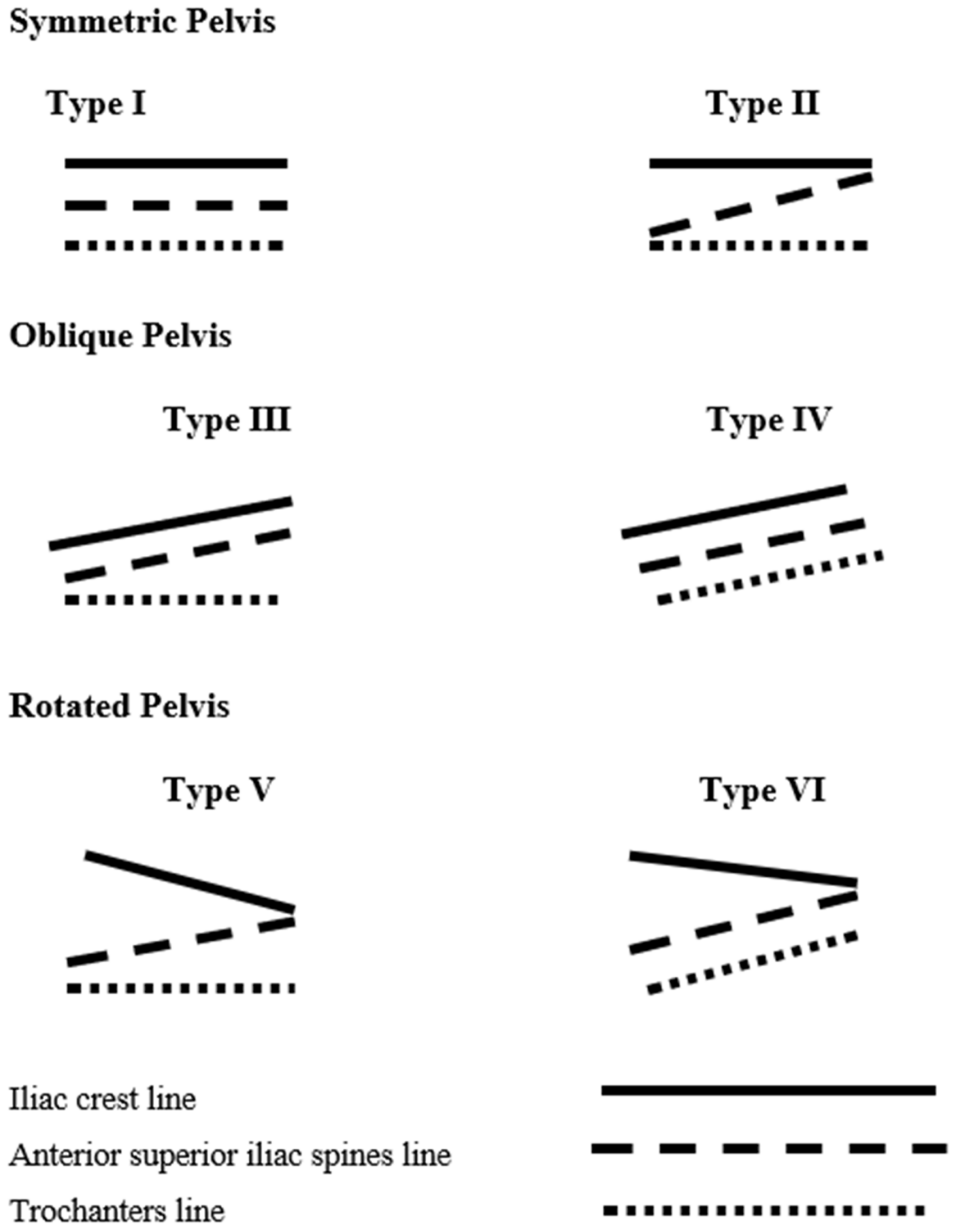
Graphic representation of pelvic asymmetry types (own source).

All the examinations were performed in the morning, to ensure the uniform measurement conditions. Every individual was tested three times and the averaged data were protocoled. All the measurements were performed by the same, experienced investigator. During each examination, the rule was followed that the examined landmark was touched with the tip of the middle finger and the measuring device arms were positioned strictly on the radial side of the middle finger. The material collected was divided into groups according to the spatial location of the selected landmarks on the pelvic girdle, using the author’s original clinical typology (Figure 2).

The proposed clinical pelvic symmetry classification (Preece et al., 2008) is based on the analysis of mutual position of lines running through the selected landmarks of the lumbo-pelvic-hip complex:

1. the line connecting the tops of the iliac crests—IC
2. the line connecting the anterior superior iliac spines—ASIS
3. the line connecting the tops of trochanter major of the femur—TM

The measurement was carried out with an accuracy of 1°.

The proposed classification by the symmetry of positioning of the selected landmarks in the pelvic girdle and lower limbs:

**I. Symmetric Pelvis (SP)**

- Type 1—All lines run parallel to the floor.
- Type 2—The iliac crest line and the trochanter major line run parallel to the floor, the position of the iliac spines may show small signs of asymmetry. This may be caused by individual anatomical differences (Duboc et al., 2015).

All study subjects with asymmetry < 1° were treated as those with a symmetrical pelvis.

**II. Oblique Pelvis (OP)**

- Type 3—The iliac crest line and the iliac spine line run the same way, in an oblique way, the trochanter line is parallel to the floor—functionally oblique pelvis (connected with, for example, asymmetrical stress of the gluteus medius).
- Type 4—The lines of the iliac crests, the iliac spines and the trochanters run the same, oblique way—structurally oblique pelvis (e.g., short lower limb).

**III. Rotated Pelvis (RP)**

- Type 5—the iliac crest line and the anterior superior iliac spine line are alternatively asymmetrical. The line of the greater trochanters runs parallel to the floor—functionally rotated pelvis.
- Type 6—the iliac crest line and the anterior superior iliac spine line are alternatively asymmetrical. The line of the greater trochanters is tilted against the floor. Most frequently in the same way as the line of the anterior superior iliac spines—structurally rotated pelvis.

Together with the quantitative characteristics of the variables, the characteristics of frequency of the occurrence of oblique and rotated pelvis, depending on the asymmetry ratio, was presented. The classification was made by means of analysis of the angle of asymmetry and determination of its intensity according to the authors’ own typology:

1. The base to determine the obliquity of the pelvis was the inclination angle of the line of the apices of the iliac crests against the horizontal axis.
2. The pelvic rotation was determined based on the angle between the line of the apices of the iliac crests and the line of the anterior superior iliac spines.

It was assumed that:

1. Slight asymmetry ≥ 1^o^ ≤ 3,0^o^ (percentile < 25).
2. Moderate asymmetry > 3^o^ ≤ 6^o^ (percentile 25–75).
3. Significant asymmetry >6^o^ (percentile > 75).

### Data analysis

The minimum size of the sample was determined based on the formula:


$$\begin{array}{l} \mathrm{Nmin}=\mathrm{NP}\;\left(\alpha 2\cdot f\left(1-f\right)\right)\mathrm{NP}\cdot \mathrm{e2}+\alpha 2\cdot f\left(1-f\right)\mathrm{Nmin}\\ =\mathrm{NP}\left(\alpha 2\cdot f\left(1-f\right)\right)\mathrm{NP}\cdot \mathrm{e2}+\alpha 2\cdot f\left(1-f\right) \end{array}$$

where Nmin, minimum sample size; NPNP, population from which the sample is selected *n* = 2,131, α—level of confidence for the results = 95%, f—fraction size = 0.5 e—maximum error assumed 5% = 0.05 (Zalewska and Niemiro, 2022).

The statistical analysis of the material was conducted using MedCalc ver.20.104 package. Distributions of the variables were determined by means of Shapiro–Wilk test. The results were presented as average and standard deviation completed with the median. The analysis of differences was performed using nonparametric tests (Mann–Whitney *U*-test). To analyze the qualitative variables, Chi-squared test was used. Relationships between the variables were investigated using Spearman’s rank correlation tests.

## Results

**Figure 3:**
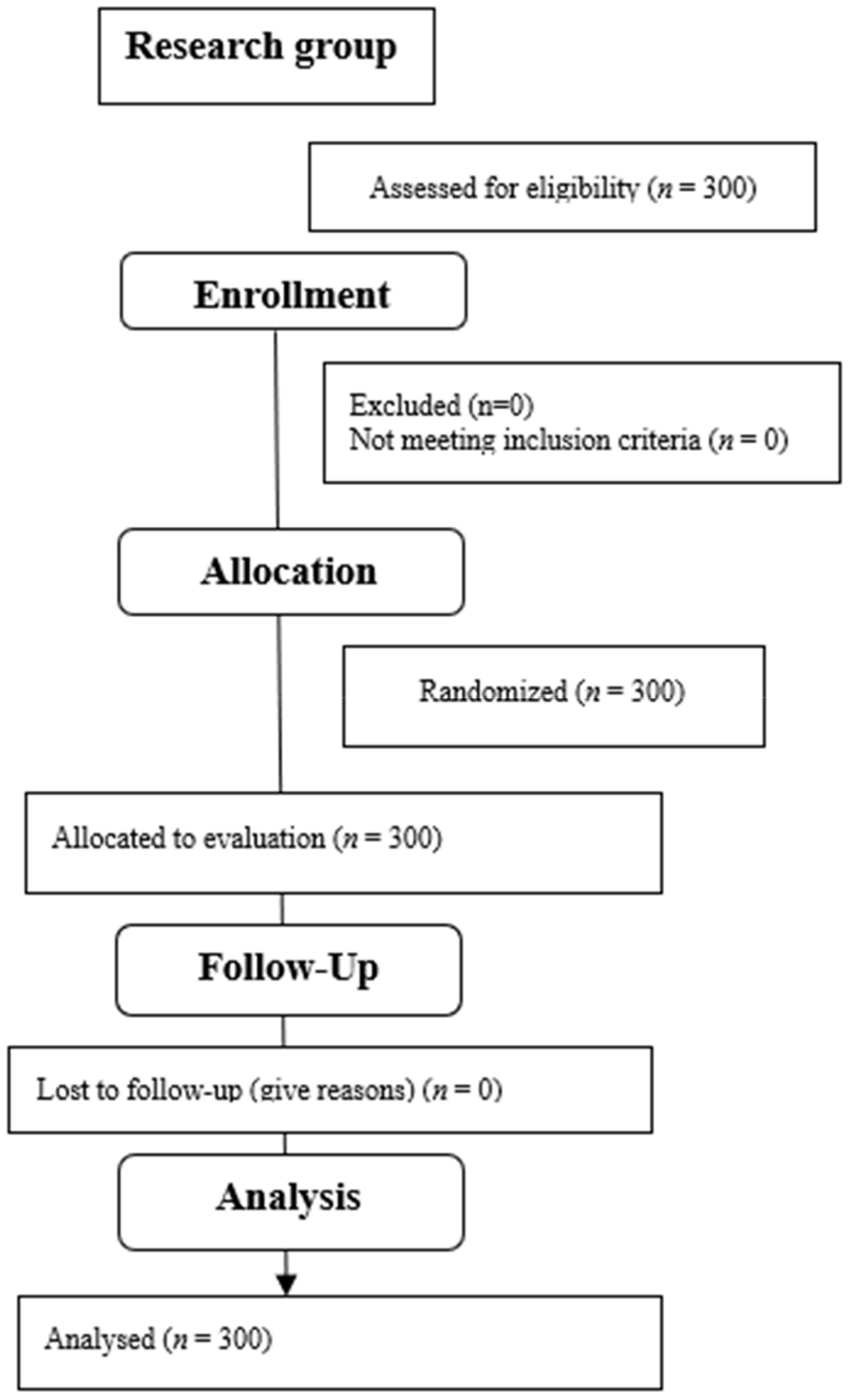
Flow diagram.

The sample consisted of 300 individuals (150 women and 150 men) aged 19–29 years. Table 1 presents the detailed anthropometric data of the sample. Figure 3 shows the qualification stage.

**Table 1:** Research group.

Sex variable	Women *n* = 150	Men *n* = 150
	Mean ± Std.	Mean ± Std.
Weight [kg]	61.5 ± 8.71	78 ± 10.69
Height [cm]	166 ± 5.99	18 ± 7.81
BMI [kgm^−2^]	21.6 ± 2.04	22.7 ± 1.35

*n*, number of participants; Std, standard deviation.

### Position of selected landmarks of the pelvic girdle and lower limbs

The analysis of symmetry between the examined landmarks shows significant differentiation depending on the gender. At the same time, individual results are clearly and significantly dispersed (Table 2; ICA *p* = 0.029; ASISA *p* = 0.020, TMA *p* < 0.001). The symmetry analysis which takes into account their direction also shows significant gender-related differences. Symmetric positioning of the iliac crests was observed in only 32% of the respondents (*p* = 0.001). The iliac crest depression on the left side was more frequently observed – in 41% of the respondents. This occurred more often in women (44%) than in men (38%). The iliac crest depression on the right side was observed in only 18% of women and as much as 36% of men (Table 3). The opposite situation was observed in terms of direction of the anterior superior iliac spine asymmetry (*p* = 0.028). The left spine was lowered in only 10% of women and men, whereas lowered right iliac spine was diagnosed in 57.7% of women and as much as 70% of men (Table 3). Symmetrical position of the spines was observed in only 33.3% of female and 20% of male respondents (Table 3). Symmetrical position of the trochanters (equal length of the lower limbs) was observed in 81.3% of women and 56% of men (*p* ≤ 0.000). Trochanter depression on the right side was observed in 14% of women and as much as 36.7% of men (Table 3).

**Table 2:** Size of iliac crest angle (ICA), anterior superior iliac spine angle (ASISA), and trochanter major angle (TMA) by gender.

Sex variable	Women *n* = 150	Men *n* = 150	*p*
	Mean ± Std. (median)	Mean ± Std. (median)	
ICA (^o^)	1.6 ± 1.72 (1.0)	2.0 ± 1.75 (2.0)	0.029^*^
ASISA (^o^)	1.8 ± 1.7 (2.0)	2.3 ± 1.72 (2.0)	0.020^*^
TMA (^o^)	0.5 ± 1.15 (0.0)	1.2 ± 1.62 (0.0)	<0.001^*^

ICA, iliac crest angle; ASISA, anterior superior iliac spine angle; TMA, trochanter major angle; *p*, *p*-value (Mann–Whitney test); *, statistically significant differences; n, number of participants.

**Table 3:** Direction of asymmetry of IC, ASIS, and TM tops position by gender.

Sex Variable	Women *n* = 150						Men *n* = 150						*p*-value test
	Left ↓		Symmetric		Right ↓		Left ↓		Symmetric		Right ↓		
	*N*	%	*N*	%	*N*	%	*N*	%	*N*	%	*N*	%	
IC	66	44	57	38,4	27	18	57	38	39	26	54	36	0.001^*^
ASIS	15	10	50	33.3	85	57.7	15	10	30	20	105	70	0.028^*^
TM	7	4.7	122	81.3	21	14	11	7.3	8	56	55	36,7	<0.000^*^

IC, top of iliac crest; ASIS, top of anterior superior iliac spine; TM, top of trochanter major; p, *p*-value (Chi^2^ test); *, statistically significant differences; ↓, landmark located below; *n*, number of participants.

### Frequency of occurrence of identified types of pelvic girdle asymmetries in young adults

The analysis of the occurrence of the certain types of pelvis shows significant differences between women and men (OP *p* = 0.010; SP *p* = 0.014; Table 4). In the group of women, the rotated pelvis was the most often observed (39.4%) asymmetry, while for men, it was the oblique pelvis (40%). Symmetric positioning of the pelvis was observed in only 22% of the respondents. It was more frequent among women (37.3%). More detailed analysis by pelvic asymmetry subtypes showed their statistical differentiation between women and men (*p* < 0.0001*). The dominating type in the symmetric pelvis group was Type 1 (Table 4). In the oblique pelvis group, Type 4 (structural) was predominant. In the rotated pelvis group, it was Type 5 (functional) with the greater trochanter tops located at the same level (Table 5).

**Table 4:** Frequency of occurrence of pelvic girdle asymmetry types in examined groups.

Sex	Women *n* = 150		Men *n* = 150		Women + Men *n* = 300		Women vs. Men *p*
Types of pelvic asymmetry	*N*	%	*N*	%	*N*	%	
OP	35	23.3	60	40	95	31.7	0.010^*^
SP	56	37.3	33	22	89	29.7	0.014^*^
RP	59	39.4	57	38	116	38.7	0.852
*p*	0.032^*^		0.012^*^				

p, *p*-value (Chi^2^ test); *, statistically significant differences; OP, Oblique Pelvis; SP, Symmetric Pelvis; RP, Rotated Pelvis; *n*, number of participants.

### Quantitative and qualitative assessment of angle of obliquity and rotation of pelvis in examined groups

**Figure 4:**
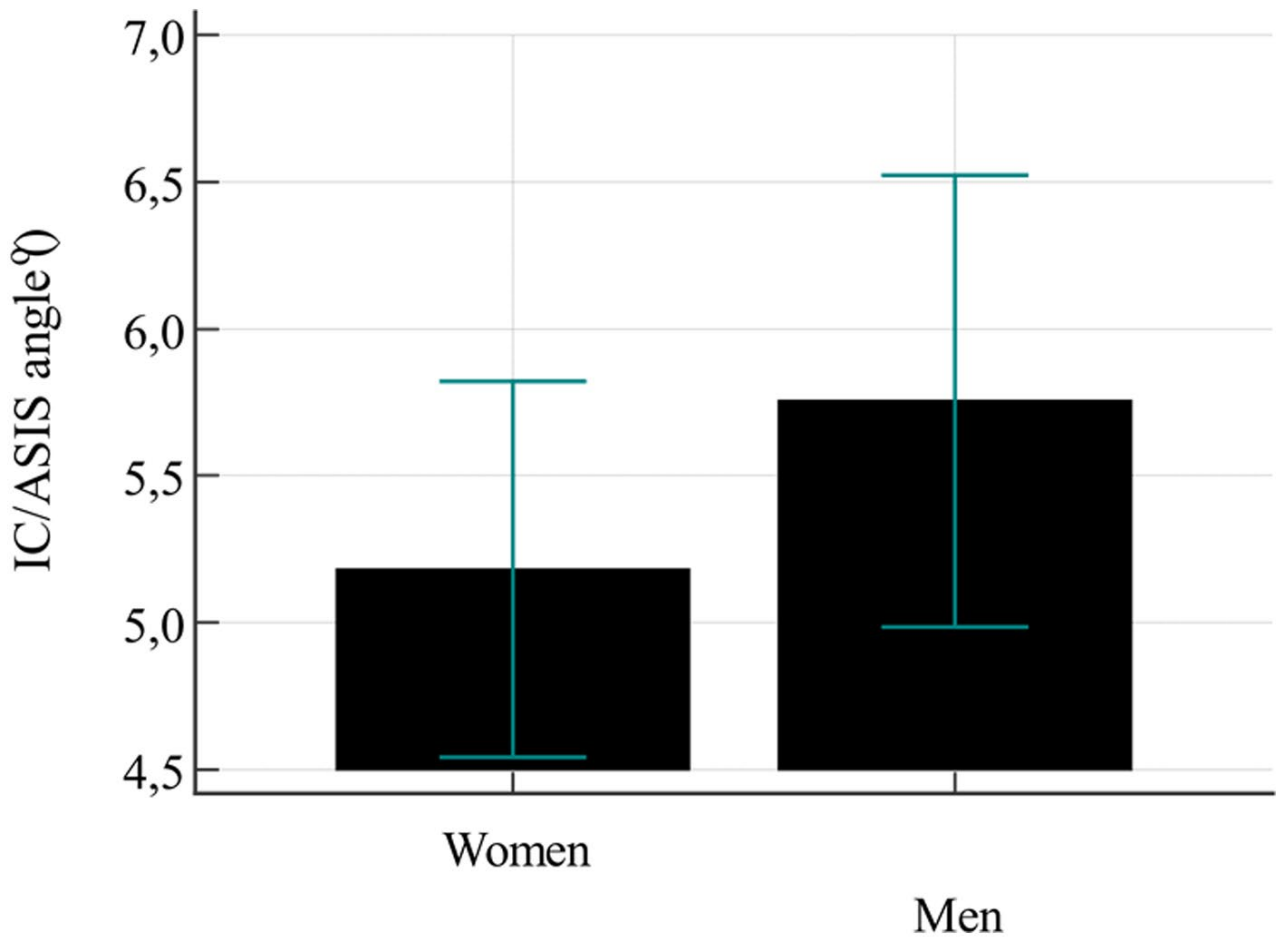
Mean value of pelvic rotation.

**Figure 5:**
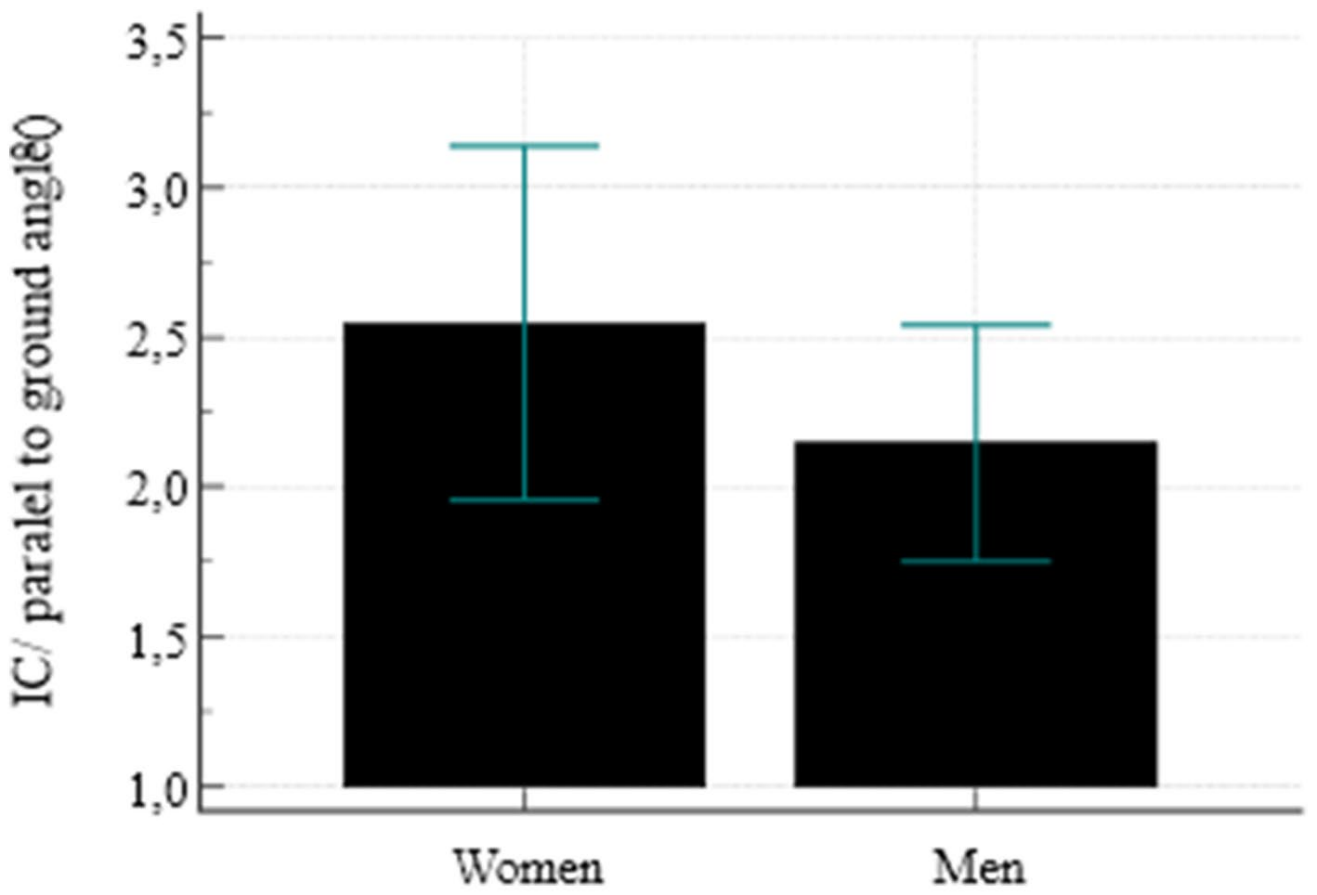
Mean pelvic obliquity value.

**Figure 6:**
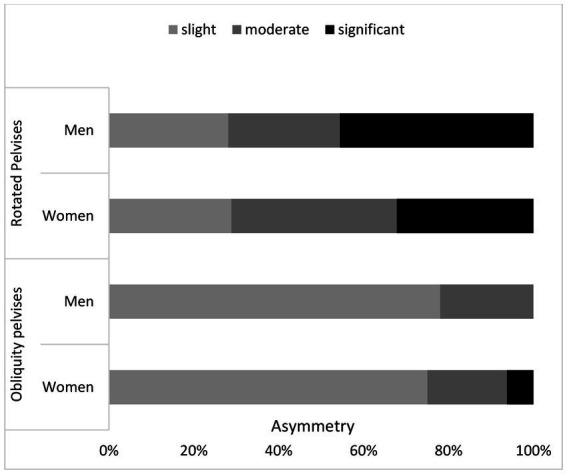
Frequency and magnitude of rotated and oblique pelvises (%).

The analysis of moderate rotation of the pelvis, measured as the angle between the line running through the tops of the IC and the ASIS, indicated that the variable distribution is different for women and for men (Figure 4). As for men, slightly higher values were reported but these differences were not statistically significant (*p* = 0.253). The opposite situation was identified during the analysis of the angle of the pelvic obliquity (Figure 5). Women, in turn, showed slightly higher mean values but here too, the differences were not statistically significant (*p* = 0.245). The analysis of frequency and angle of pelvic asymmetries did not reveal any significant differences between male and female respondents (oblique pelvises *p* = 0.309, rotated pelvises *p* = 0.594). However, it was noticed that in the case of oblique pelvises, slight asymmetries dominated in both groups, whereas in the rotated pelvis group—moderate and significant asymmetries were predominant (Figure 6).

### Assessment of correlations between variables

The analysis of correlations between the body mass and height and the asymmetry angle of the iliac crests, the anterior superior iliac spines and the greater trochanters, conducted using Spearman’s correlation coefficient, did not show linear correlations between the investigated parameters. Minor correlations between the body mass and the angle of trochanter major asymmetry were considered accidental (Table 6).

**Table 5:** Differences in frequency of occurrence of pelvic girdle asymmetry types depending on gender.

Types of pelvic asymmetry		Women		Men		Women + Men	
		*N*	%	*N*	%	*N*	%
SP	Type I	49	32.7	26	17.3	75	25
	Type II	6	4	7	4.7	13	4.3
OP	Type III	11	7.3	6	4	17	5.7
	Type IV	24	16	54	36	78	26
RP	Type V	58	38.7	47	31.3	105	35
	Type VI	2	1,3	10	7.7	12	4

OP – Oblique Pelvis; SP – Symmetric Pelvis; RP – Rotated Pelvis; n – number of participants

**Table 6:** Assessment of correlations between anthropometric variables and angle of asymmetry of selected pelvic girdle landmarks.

Variables	Sex	IC		ASIS		TM	
		rho	*p*	rho	*p*	rho	*p*
Weight	♀	0.073	0.373	0.048	0.561	0.177	0.030^*^
	♂	−0.011	0.896	−0.107	0.191	−0.145	0.077
Height	♀	0.143	0.082	0.086	0.293	0.126	0.373
	♂	−0.140	0.088	−0.115	0.161	0.084	0.304

IC, top of iliac crest angle; ASIS, anterior superior iliac spine angle; TM, top of trochanter major angle; rho, Spearman Correlation Coefficient; ♀, Women; ♂, Men; *, statistically significant differences.

## Discussion

The aim of the study was to present the results of inclinometer measurements of selected landmarks in the lumbo-pelvic-hip complex in young adults aged 19–29. The analysis of occurrence of spatial pelvic asymmetry was based on the authors’ original, clinical classification and the significance of the body mass and height for the analyzed asymmetries. The analysis of positions of the selected landmarks of the pelvic girdle shows frequent occurrence of asymmetries. The iliac crest asymmetry was observed in 68% of the respondents, women slightly more often than men. The iliac crest depression on the left side was more frequently observed, similar in both groups. Greater differences occurred in the lowering of the iliac crest on the right side. Greater asymmetry was found in men. Such a high percentage of iliac crest asymmetry confirms the previous observations of the authors as well as other researchers (Brady et al., 2003; Kurki, 2017; Mundorf et al., 2021; Zhang et al., 2022a). ASIS asymmetries were observed equally often. Contrary to the iliac crests which were more often lowered on the left side, the iliac spines were more frequently lowered on the right side. The results of the analysis of trochanter major tops symmetry were very different. More symmetrical alignment was observed in women. According to the authors, this may indicate a significant percentage of functional asymmetries or asymmetries which are not related to the limb inequality. On the other hand, bilateral differences in bone length of the upper and lower limbs are associated with different mechanical stress put on the bones during growth and are called directional asymmetry. In the upper limbs, this skeletal asymmetry is usually visible on the dominant side, while in the lower limbs—on the opposite side. This is probably due to auxiliary contractions of the opposite muscles, which affect the bone growth. This contralateral dominance in the upper and lower limbs is known as cross symmetry pattern (Alqadah et al., 2018). In the study described herein, only individuals with the right-sided dominance in the area of upper and lower limbs were examined and the connection with the crossover symmetry in the pelvic area was not clear.

Asymmetry of the pelvis exhibited as the difference in the height of the right and left hip is called pelvic obliquity or leg length inequality (Zhang et al., 2022b). According the authors, these terms are not synonyms due to different causes of the asymmetries. Not every difference in the iliac crest height results from unequal limb length. Considering only the asymmetry in the iliac crest height and making therapeutic decision based on that may affect the effectiveness of the therapy. This was taken into consideration when the pelvis asymmetry classification used in the study was developed. The analysis conducted showed that only one third of the respondents had symmetric pelvis. Also, symmetric pelvis was found significantly more often in women. Oblique pelvis was observed significantly more frequently in men. In turn, rotated pelvis was observed more often in female participants.

In the group of individuals with oblique pelvis, Type 4 (structural) of the asymmetry was definitely dominating. Among the respondents with rotated pelvis, Type 5 (functional) was predominant as compared to Type 4 (structural) where, together with the contralateral asymmetry of the iliac crests and the iliac spines, the trochanter major asymmetry was observed. There was a minor differentiation of the mean pelvic obliquity angle and rotation angle between women and men but the differences were not statistically significant. The qualitative analysis of the asymmetry angle showed that women and men with oblique pelvis had mainly slight asymmetries while individuals with rotated pelvis – moderate and significant asymmetries. It was concluded that anthropometric parameters had no linear correlations with the asymmetries studied.

Body asymmetry is more of a standard rather than an exception. Thus, the occurrence of different types of is are not surprising. This refers both, to structure and function. Starting from brain and central nervous system asymmetries (Kanchan et al., 2008; Medina-Rivera, 2016; Güntürkün and Ocklenburg, 2017). They can have many causes, for example optic nerve diseases (Traver-Vives et al., 2021) or differences in lateralization ontogenesis (Kanchan et al., 2008), craniofacial asymmetries (Maloney, 2019) or ontogenetic differences in the limb length (Alqadah et al., 2018; Bishop et al., 2018). The relations between the structural and functional asymmetries are also often analyzed (Brady et al., 2003; Applebaum et al., 2021). Asymmetries are also assessed in the context of athletic achievements. The sports experience of the players (Piepiora et al., 2022a), their sports level (Piepiora et al., 2021) and anthropometric predispositions (Piepiora et al., 2022b) may not be without significance in this matter. It is commonly assumed that bilateral asymmetries have negative effect on sports results, however studies do not confirm such relationship fully (Betsch et al., 2012; Allam and Schwabe, 2013). The area of the pelvic girdle is of special interest due to its complex role in the functioning of humans. Surely, one of the important issues is the effect of correctly aligned pelvic girdle in the spine position (Holmes et al., 2019). Each incorrect position of the pelvis requires back compensation and is a challenge to the control the whole balance process (Pitkin and Pheasant, 1936; Juhl et al., 2004). Pelvic asymmetry is connected with directional biomechanical load (Kurki, 2017). One of the first publications on measuring the position of the pelvis was the work by Pitkin and Pheasant (1936) and Gordon and Davis (2019). They were the first to use the inclinometer measurements to examine the pelvis and analyze the relationship between limb length difference and pelvic rotation. Their first classification of pelvic asymmetries was based on the analysis of radiological images made by Lloyd and Eimbrink. Their system has never been published but only used for educational purposes in the Philadelphia College of Osteopathic Medicine in the early 1950s. The research confirming the connections between pelvic asymmetries and therapeutic practice was validated by Heilig (1978) and Vogt et al. (2020). Gnat and Bialy (2015) proposed an interesting assessment of the pelvic asymmetries. Their method enables the assessment of both asymmetry in the frontal plane and its rotation. However, it seems rather time-consuming and does not enable—in the authors’ opinion—full differentiation between functional and structural asymmetries. This is a limitation to therapeutic decision-making process. The measuring method includes the asymmetry of the hip to a small extend only (Duboc et al., 2015).

The method of assessing the pelvic symmetry was to be used for initial differentiation of functional and structural asymmetries in the pelvic area. In the right conditions, the lines of the tops of the iliac crests, the anterior superior iliac spines and tops of the trochanter major should run parallel to the floor. With the anthropometric leveler Duometer, not only quantitative but also qualitative data could have been obtained (Preece et al., 2008).

### Study limitations

The authors are fully aware that all measurements of the location of bone points on the human body can sometimes introduce an illusory sense of accuracy and reliability. This is due to the fact that the examined bone points are not examined directly but through the body tissues. This applies to most anthropometric studies, even those using advanced methods of three-dimensional analysis. Such an assessment is particularly disturbed by palpation. The use of different measuring instruments reduces the risk of making a mistake, but does not completely eliminate it. Any measurement other than X-ray will always have some error related to the construction of the human body.

During the research, the authors tried to maintain restrictive test conditions to maintain the reliability and repeatability of measurements. Previous studies have shown that measurements using the Duometer show high repeatability under the conditions of tests conducted by an experienced examiner (Bibrowicz and Bibrowicz, 2011a; Bibrowicz et al., 2022).

The results presented herein are a part of a wider research program and are naturally limited to the descriptive analysis of asymmetries. Other aspects of the study, connected with pelvic asymmetry influence on the health, balance, posture quality or other detailed correlations will be presented in the next reports.

## Conclusion

1. Asymmetries in the pelvis area are common; they were observed in less than three-quarters of the examined population.
2. Oblique pelvis was found in less than a quarter of women and in more than one-third men with the predominant structural asymmetries.
3. Rotated pelvis was observed in more than one-third of women and men with dominating functional asymmetries.
4. There were no linear correlations between the body mass and height, and the angle of asymmetries.

## Data availability statement

The original contributions presented in the study are included in the article/supplementary material, further inquiries can be directed to the corresponding author.

## Ethics statement

The studies involving human participants were reviewed and approved by the Bioethics Board of the Higher School of Physiotherapy in Wroclaw, approval No. 1/2010 of 10.04.2010. The patients/participants provided their written informed consent to participate in this study. Written informed consent for participation was not required for this study in accordance with the national legislation and the institutional requirements.

## Author contributions

KB contributed to the conception and design of the study and organized the database. KB, TS, KO-C, BG-W, PK, and ZH performed the statistical analysis, wrote the first draft of the manuscript, and wrote sections of the manuscript. All authors contributed to the article and approved the submitted version.

## Funding

Publication partly financed by the Ministry of Science and Higher Education under the 2019–2022 Regional Initiative of Excellence programme, project number: 022/RID/2018/19, grant amount: PLN 11.919.908.

## Conflict of interest

The authors declare that the research was conducted in the absence of any commercial or financial relationships that could be construed as a potential conflict of interest.

## Publisher’s note

All claims expressed in this article are solely those of the authors and do not necessarily represent those of their affiliated organizations, or those of the publisher, the editors and the reviewers. Any product that may be evaluated in this article, or claim that may be made by its manufacturer, is not guaranteed or endorsed by the publisher.
